# Gendered Pathways: How Mathematics Ability Beliefs Shape Secondary and Postsecondary Course and Degree Field Choices

**DOI:** 10.3389/fpsyg.2017.00386

**Published:** 2017-04-06

**Authors:** Lara Perez-Felkner, Samantha Nix, Kirby Thomas

**Affiliations:** ^1^Department of Educational Leadership and Policy Studies, Center for Postsecondary Success, Florida State UniversityTallahassee, FL, USA; ^2^Department of Sociology, Florida State UniversityTallahassee, FL, USA

**Keywords:** STEM, gender, sex segregation, STEM education, college majors, ability beliefs, mathematics ability, challenge

## Abstract

Do mathematics ability beliefs explain gender gaps in the physical science, engineering, mathematics, and computer science fields (PEMC) and other science fields? We leverage U.S. nationally representative longitudinal data to estimate gendered differences in girls' and boys' perceptions of mathematics ability with the most difficult or challenging material. Our analyses examine the potentially interacting effects of gender and these ability beliefs on students' pathways to scientific careers. Specifically, we study how beliefs about ability with challenging mathematics influence girls' and boys' choices to pursue PEMC degrees, evaluating educational milestones over a 6-year period: advanced science course completion in secondary school and postsecondary major retention and selection. Our findings indicate even at the same levels of observed ability, girls' mathematics ability beliefs under challenge are markedly lower than those of boys. These beliefs matter over time, potentially tripling girls' chances of majoring in PEMC sciences, over and above biological science fields, all else being equal. Implications and potential interventions are discussed.

## Introduction

Over recent decades and across countries, women have been surpassing men in college enrollment and degree attainment, with the exception of a narrow set of persistently male-dominated mathematics-intensive degree fields (Hill et al., 2010; Charles, 2011b; DiPrete and Buchmann, 2013). Women are particularly underrepresented in physical, engineering, mathematics, and computer (PEMC) sciences (Perez-Felkner et al., 2012; Schneider et al., 2015). Such **sex segregation** (see Reskin, 1993) in undergraduate majors has two important consequences. First, studies show these disparities contribute considerably to the gender pay gap, with notable implications for women and the families they often support (Bobbitt-Zeher, 2007). Second, PEMC fields not only have some of the smallest gender gaps in pay (Corbett and Hill, 2012) but also align with areas of growth in our increasingly scientific global economy (National Science Foundation, 2016). Women are projected to comprise nearly 60% of university students by 2025 but earn a clear minority of PEMC undergraduate degrees (National Science Foundation and National Center for Science and Engineering Statistics, 2015), a pattern that does not have an end in sight (OECD, 2012).

KEY CONCEPT 1 **Sex segregation.**

Sex segregation refers to the tendency of occupations to be held by men or women, and perceived as most appropriate for one or the other. The sex typing of jobs has consequences for earlier decisions: (a) students' corresponding choice of undergraduate degree fields and (b) course work and ability beliefs prior to postsecondary school.

Our recent paper demonstrated the role of mathematics **ability beliefs** in girls' abstention, retention, and attrition from PEMC fields, during the period when most talented young women tend to depart from these career pathways: between secondary school and the early years of postsecondary school (Berryman, 1983; Morgan et al., 2013).

KEY CONCEPT 2 **Ability beliefs.**

Ability beliefs are individuals' perceptions of the nature and level of their academic ability, including their mathematics ability.

Leveraging the most recent and complete U.S. panel of data available, the Education Longitudinal Study of 2002/12 (ELS), we investigated how girls' and boys' ability-related beliefs influenced their subsequent choice of postsecondary degree fields. In particular, we estimated the intersecting relationship between gender and **perceived ability under challenging conditions** on each subsequent step on the pathway to PEMC undergraduate degrees: completing advanced high school science courses, persistence in a major, and major selection (Nix et al., 2015). This focused review discusses key findings from this longitudinal study as well as additional analyses expanding on our results and their implications. New findings further distinguish the particular influence of gendered differences in perceived ability under challenge, holding constant objective measures of mathematics ability.

KEY CONCEPT 3 **Perceived ability under challenge.**

Students' assessments of their ability to complete work or understand concepts that they believe is the most difficult or advanced in a specific domain of study.

## Women's participation in science: high school through college

Research explaining women's underrepresentation in the scientific labor force tends to be bifurcated into K–12/childhood and higher education/adulthood categories. The marriage of longitudinal data and a comprehensive framework can be elusive. The dominant literature continues to argue for a “pipeline” to STEM fields, suggesting that young women move sequentially from taking secondary school courses to declaring majors and graduating with undergraduate degrees, into graduate school, and into the scientific community. This linear model appears overly simplistic, especially for less socioeconomically advantaged students (Goldrick-Rab, 2006) and women of color in STEM fields (Reyes, 2011). And yet, there are clear steps from high school through the college years that are critical to preventing talent loss among potential female scientists (e.g., Morgan et al., 2013).

Secondary school experiences can influence gendered differences in who majors in postsecondary science fields. Even highly able girls often do not take the most advanced mathematics courses and subsequently tend to pursue non-PEMC major fields (Riegle-Crumb et al., 2006; Perez-Felkner et al., 2012). Taking physics and calculus increases girls' chances of entering PEMC and related postsecondary majors (Riegle-Crumb et al., 2006), therefore it seems promising that girls' completion of advanced mathematics courses has increased in recent years (Dalton et al., 2007; DiPrete and Buchmann, 2013). Nevertheless, the gender gap in PEMC has not disappeared (Riegle-Crumb et al., 2012); indeed, in some fields, like computer science, women's representation among undergraduate majors has declined (Corbett and Hill, 2015).

Across the globe, the lack of gender parity in high-growth and high-earning PEMC fields may have broader implications for women's economic futures. Notably, women tend to select degree fields with some of the lowest median earnings (Carnevale et al., 2011). Gendered variation in undergraduate field of study is a principal driver of income inequality, both indirectly through subsequent occupational choices and even directly, independent of work-related factors (Bobbitt-Zeher, 2007). Women are the primary earners for over 40% of U.S. households with children (Wang et al., 2013). Thus, the implications of the degree fields they choose and subsequent returns for their education have consequences for the lives of women, families, and societies, especially those historically underrepresented in higher education and with fewer socioeconomic advantages. U.S. women earn 78 cents on the dollar as compared to men—worse still for women of color (American Association of University Women (AAUW), 2015). Importantly, the gender gap in earnings is smaller in PEMC fields; while few in number, women computer programmers earn 92% as much as their male peers (Corbett and Hill, 2015). This study informs our understanding of how gender shapes degree field choice in the context of a global pattern of rapidly expanding university participation—including in the U.S.—in which women of all groups have experienced educational gains in nearly all areas except PEMC degrees.

## Ability beliefs and their consequences

Decades of research have shown students' beliefs about their ability—especially in mathematics—can have longer-term implications. Girls are often socialized to associate scientific careers with men (Lee, 1998; Cheryan, 2012), and engage less often with mathematical and scientific tasks during adolescence (Eccles, 1994; Eccles and Wigfield, 2002), a finding corroborated across diverse populations (Watt, 2006; Else-Quest et al., 2013). A nationally representative study of U.S. adolescents in the early 1990s showed girls assess their mathematics ability more negatively than do boys, with consequences for their later career decisions (Correll, 2001). These negative self-assessments seem to be a response to the sex-typed associations alluded to above (see also Correll, 2004; Cheryan and Plaut, 2010), which are common in many industrialized nations (Charles and Bradley, 2009; Charles, 2011a). Experimental social psychologists have demonstrated that girls and women often experience **stereotype threat** in challenging mathematics contexts (Spencer et al., 1999; Good et al., 2003). The negative consequences of these stereotypes may be exacerbated for those who have a fixed rather than **growth mindset** about their mathematics ability (Dweck, 2006). Indeed, girls *and* boys are more likely to declare PEMC undergraduate majors when they have more positive orientations to mathematics, including perceived mathematics ability and growth mindset (Perez-Felkner et al., 2012). Even during college, positive beliefs about one's mathematics ability—more commonly held by men—are associated with continuing on to complete majors in PEMC and related fields (Sax, 1994; Sax et al., 2015). All together, these studies indicate how mathematics ability beliefs affect women's pathways toward scientific degrees.

KEY CONCEPT 4 **Stereotype threat.**

Stereotype threat is the experimentally demonstrated phenomenon showing individuals tend to underperform when negative stereotypes about their identity group are made salient. For example, when women are primed with a reminder of men's perceived dominance in mathematics, they perform lower on academic tests than do otherwise similar women who did not experience this stressor.

KEY CONCEPT 5 **Growth mindset.**

Individuals with a growth mindset believe mathematics ability is malleable, that it can be learned, as compared to those who believe it is a fixed trait inherited at birth and irresponsive to effort.

Our study is particularly interested in perceived ability when encountering *challenge*, for three primary reasons. First, underrepresented individuals may face particular personal challenges when breaking into a field where they in the minority (e.g., women in PEMC)—including stereotype threat (Beilock, 2008). Second, beliefs about ability in difficult/challenging mathematics bears particular importance to success in mathematics-intensive endeavors. While previous scholars have argued for (Correll, 2001, 2004) and against (Riegle-Crumb and King, 2010) the importance of mathematics self-assessments of ability in the scientific gender gap, these and other studies have not directed attention to the specific issue of mathematics challenge. By contrast, we focus on questionnaire items indicating students' perceptions of their ability to learn and master the most difficult mathematical concepts. Previous work focused on broader perceptions of talent in mathematics as compared to verbal domains (Correll, 2001)^1^. Our approach narrows the focus from students' mathematical ability beliefs *in general* to these beliefs *in the context of the most challenging material*. Notably, girls believing mathematics ability can grow continue to engage in difficult mathematics tasks as compared to those with fixed mindsets, who may adopt a fatalistic response and withdraw from mathematics-intensive pursuits (Dweck, 2007; Good et al., 2012). Third, despite its insignificant explanatory power (Hyde, 2014), those arguing for a biological dimension to the gender gap in science focus on the upper tail of the distribution of mathematics ability—involving mastery of the most difficult mathematics problems (Hedges and Nowell, 1995; Summers, 2005). We hypothesize girls' and boys' pathways to scientific degrees can be predicted by their perceived ability under challenge, and that the nature of this relationship varies by gender.

## Methodology

### Research questions and hypotheses

Our recent study (Nix et al., 2015) and the new analyses we report on below respond to our central research question: How do girls' and boys' mathematics ability beliefs relate to subsequent steps on students' pathways to mathematically-intensive scientific majors, and how does this relationship vary by gender? Figure S1 represents our hypothesis that gender moderates the relationship between mathematics ability beliefs and pursuit of PEMC degrees. Because ability beliefs can be specific to particular task and subject domains (Csíkszentmihályi and Schneider, 2000; Bandura et al., 2001), we distinguish between perceptions of mathematics, verbal, and general ability under challenge.

### Procedures

We used nationally representative Education Longitudinal Study (ELS) restricted-use panel data collected by the U.S. National Center for Education Statistics. The base-year sample in 2002 includes (totals rounded for compliance with restricted- use data procedures) 16,200 10th graders from 750 high schools across the United States, as well as their parents, teachers, and school administrators. Follow-ups were conducted in 2004 (12th grade), 2006 (2 years after high school), and 2012 (Ingels et al., 2014). For clarity, we discuss the data primarily in reference to participants' educational stage. This study was conducted in accordance with Florida State University's Human Subjects Review Board and in full compliance with the U.S. Institute for Education Sciences Restricted Data Use procedures. Detailed technical information is provided in the Supplementary Material about our analytic samples, variables, and models. Our analysis includes three waves of panel data and represents the college-going population of U.S. students who were tenth graders in the spring of 2002 and enrolled in in degree-granting postsecondary programs by two years after high school. Our analytic sample follows 4,450 U.S. students from sophomore year of high school through declaration of a college major. We adjust for stratification in the sampling design with complex survey weighting techniques, discussed in greater depth in the Supplementary Material.

Section How Different Are Girls' and Boys' Mathematics Ability Beliefs? describes the results of regression models assessing the relationship between gender, growth mindset, and mathematics perceived ability under challenge. *Growth mindset* was calculated from a single questionnaire item asking about students' level of agreement with a statement that most people can learn to be good at math. *Mathematics perceived ability under challenge* measures indicate students' level of agreement with the following statements: “I'm certain I can understand the most difficult material presented in math texts,” “I'm confident I can understand the most complex material presented by my math teacher,” and “I'm certain I can master the skills being taught in my math class,” in the 10th and 12th grades. This analysis is further explored by including interaction terms measuring the relationship between each of these three ability belief measures and gender. In Section Do Ability Beliefs Influence Girls' and Boys' Scientific Course Completion in Secondary School?, we explain the results of ordinal logistic regression models of the highest high school science course taken, estimating the effects of gender and 10th grade ability belief measures (mathematics, verbal, and general) along with the following control variables: race/ethnicity; parent education; family income; 10th grade mathematics and verbal ability and grade point average; region; and urbanicity. In Section How Do Ability Beliefs Influence Girls' and Boys' Intended and Declared Postsecondary Majors?, we add 12th grade mathematics ability belief measures, advanced science course completion, and postsecondary institutional selectivity as controls, and include interaction terms measuring the joint effect of gender and mathematics ability beliefs on retention in PEMC majors.

## Results

### How different are girls' and boys' mathematics ability beliefs?

The 2015 study began by examining gender differences in ability beliefs. To do this, we compared girls' and boys' average ability perceptions: in mathematics, verbal, and general domains. We measured statistical differences by gender with Adjusted Wald Tests. Ability beliefs varied by gender only in mathematics, with boys notably higher in their ratings than girls. Perceived ability under challenge was measured in 10th and 12th grades and indicates students' level of agreement with the following statements: “I'm certain I can understand the most difficult material presented in math texts,” “I'm confident I can understand the most complex material presented by my math teacher,” and “I'm certain I can master the skills being taught in my math class.” Girls and boys differed most widely on 10th grade perceptions of their mathematics ability under challenge: boys rated their ability 27% higher than did girls (about 0.40 standard deviations). In addition, girls were about 0.20 standard deviations lower than boys on growth mindset (11% difference) and 12th grade perceived ability under challenge (13% difference).

In this manuscript, we examined these differences further. The results described above demonstrate gender differences in students' mathematics ability beliefs without taking objective measures of ability into account. Subsequently, we assessed how these beliefs vary across students' 10th grade mathematics ability test scores (*bytxmirr*), a measure widely used in research using ELS (e.g., Riegle-Crumb and Humphries, 2012). Two-sample *t*-tests (allowing for unequal variances) were used to statistically compare girls' and boys' ability beliefs, on average, among students at the 10th, 30th, 50th, 70th, and 90th percentile of observed ability (among students in our analytic sample, across gender). Further, we report and show gender differences in these beliefs across the distribution of observed ability.

**Figure 1:**
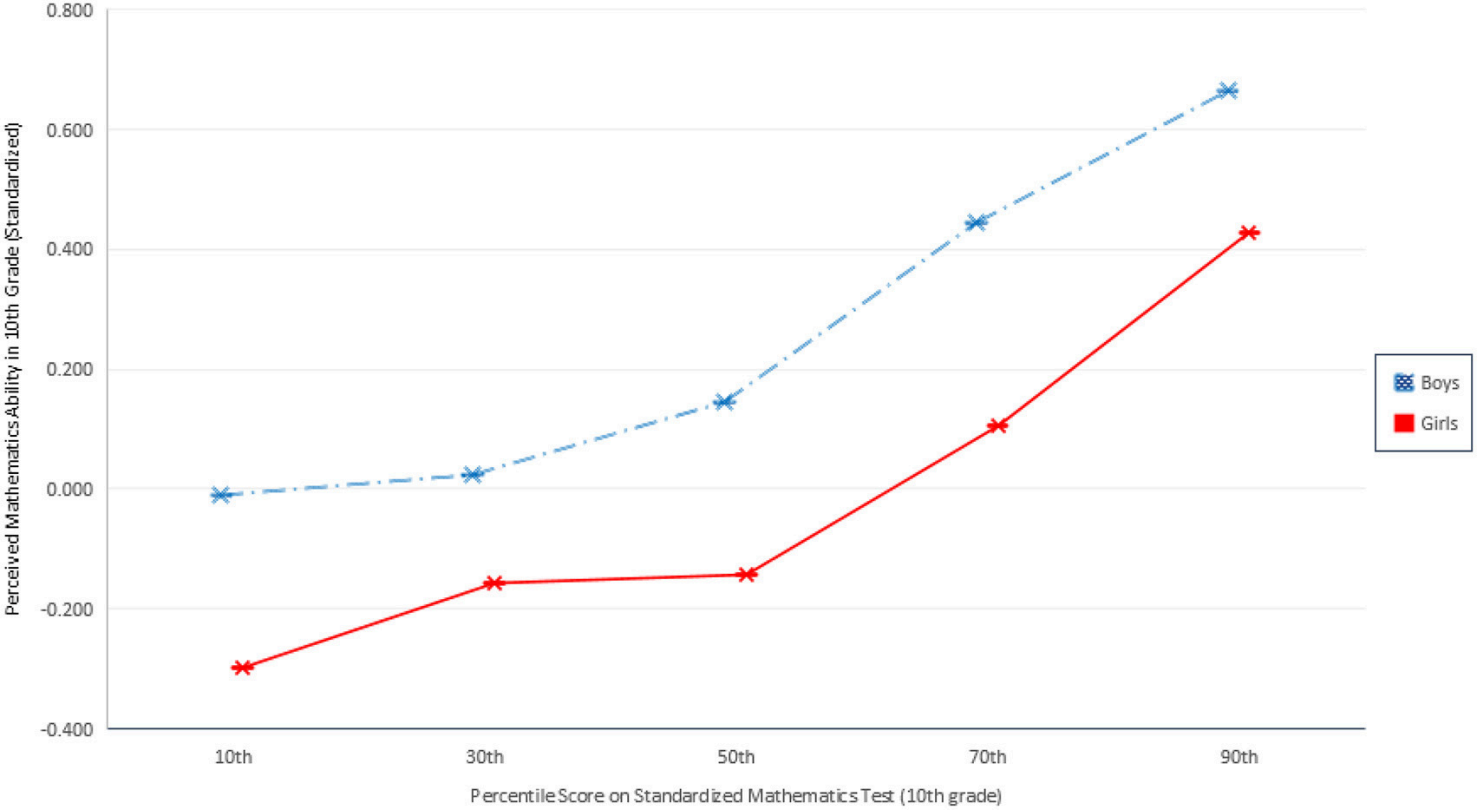
**Perceived mathematics ability in 10th grade given objective ranking of mathematics ability**.

**Figure 2:**
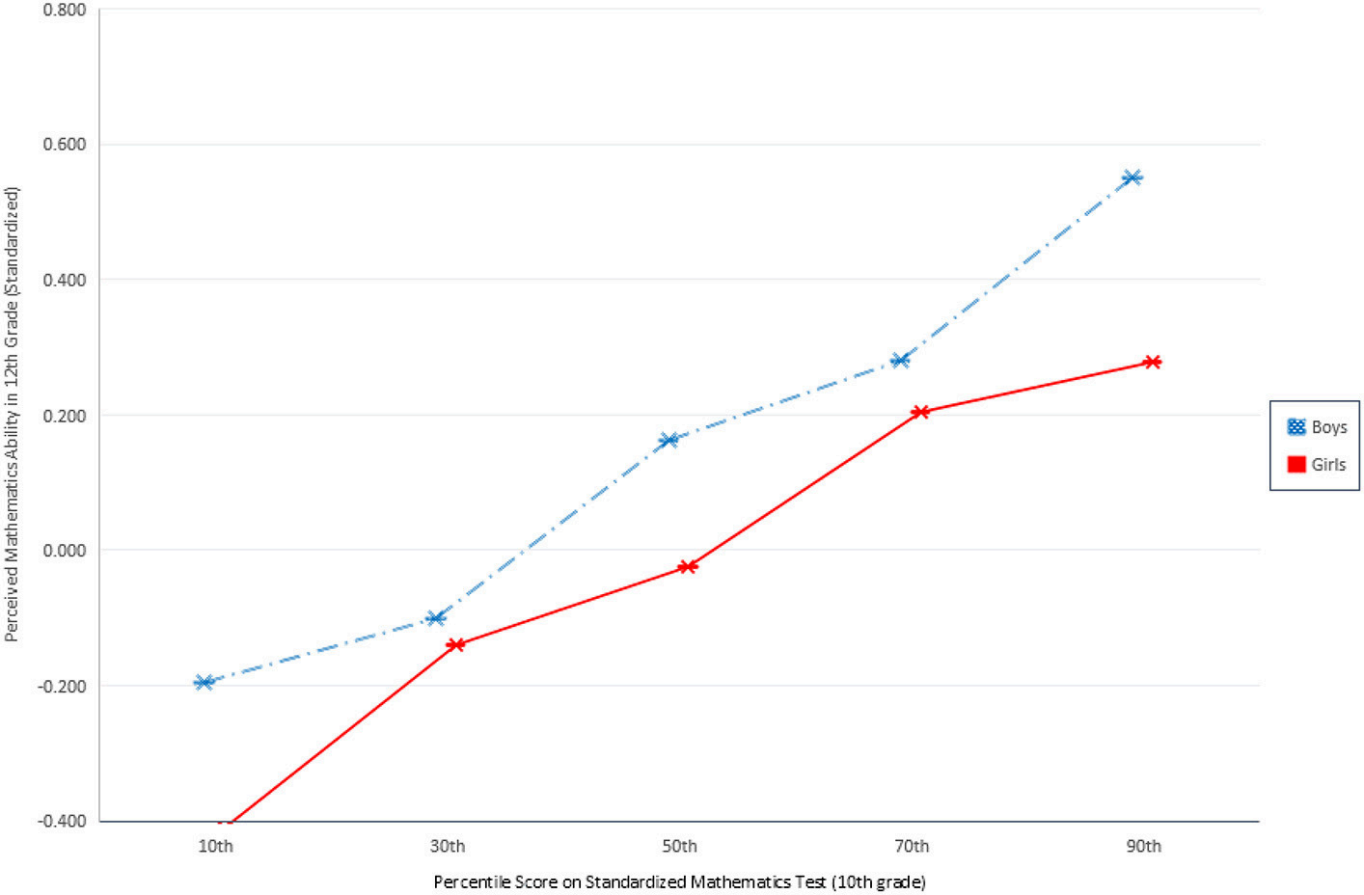
**Perceived mathematics ability in 12th grade given objective ranking of mathematics ability**.

As shown in Figures 1 and 2, gender differences in perceived mathematics ability in 10th and 12th grades are highly significant (*p* < 0.001). Across both figures, boys consistently rate their ability more highly than girls do, irrespective of their actual observed ability. The blue dashed line represents boys; girls are represented in red. The largest gender differences are found in 10th grade perceived mathematics ability under challenge. Figure 1 indicates the gender gaps are largest among the least and most talented mathematics students. Girls rate their ability in difficult mathematics systematically lower than boys: the gender difference is 0.29 standard deviations for those at the 50th percentile of observed ability, 0.34 standard deviations for those at the 70th percentile, and 0.24 for those at the 90th percentile. In sum, the gender difference in perceived ability under challenge is wide and demonstrable across all observed ability values, including, most importantly, among those students at the highest levels of ability who demonstrate the highest potential for future careers in mathematics and science. Figure 2 shows a more modest but still highly significant gender difference in perceived ability under challenge (in 12th grade) across the observed ability distribution. Notably, the widest gender difference here is at the top of the ability distribution. Whereas among girls in the 30th through 70th percentiles of observed ability fall between 0.05 and 0.19 standard deviations below boys, at the 90th percentile, 12th grade girls' perceived ability with challenging mathematics is 0.27 standard deviations lower than their male peers. In other words, *boys are significantly more confident in challenging mathematics contexts than otherwise identically talented girls*. The analyses that follow evaluate the extent to which students' ability beliefs influence students' pursuit of scientific careers in secondary and postsecondary school, and the effect of gender on this relationship.

### Do ability beliefs influence girls' and boys' scientific course completion in secondary school?

This 10th grade gender gap in perceived ability is perhaps even more consequential than at 12th grade, because perceived ability under challenge in 10th grade is a particularly strong predictor of students' decisions to take subsequent science courses in secondary school, as we showed in the 2015 paper (Nix et al., 2015). There, we used ordered logistic regressions to predict the highest science course taken in high school. Aside from students' background characteristics, and objective ability measures (model details described in Section Methodology and supplement), 10th grade perceived mathematics ability under challenge was the most influential predictor of advanced science course completion. Moreover, holding perceived math ability under challenge, objective math ability and other factors constant, girls have 24.0% lower odds than boys of completing the most advanced science courses, all else being equal.

Above and beyond the individual effects of gender and ability beliefs on advanced science course completion, we also tested whether the relationship between ability beliefs and students' chances of completing science courses varied by gender, and found that they did not. The influence of ability beliefs on students' chances of completing science courses did not differ by gender, indicating these beliefs positively influence course completion similarly for both men and women. Notwithstanding, the significant gender differences in ability beliefs and direct effect of gender on completing advanced scientific coursework suggest gender may moderate the relationship between mathematics ability beliefs and course completion.

### How do ability beliefs influence girls' and boys' intended and declared postsecondary majors?

Moving farther along girls' and boys' pathways to scientific degrees (to 2 years after high school), we examined the association between ability beliefs and gender with (A) postsecondary major retention and (B) specific major choice. Our major retention analyses compared intended postsecondary majors to declared majors, distinguishing among those who entered, stayed, and left the natural sciences (PEMC and biological science fields) with abstainers (those never indicating an interest in these fields)^2^. To evaluate students' specific major choices, we estimated these relationships with multinomial logistic regression equations comparing students' chances of majoring in PEMC, biological sciences, health sciences, and social/behavioral and other sciences to selection of majors outside of science, technology, engineering, and mathematics (STEM) fields.

Perceived mathematics ability under challenge clearly predicted retention in the natural sciences (PEMC/biology) as compared to other majors. Again, 10th grade mathematics ability under challenge is the most influential of these ability beliefs, even when including a 12th grade measure of this same indicator to capture potential change in students' mathematics ability beliefs over time. Because of this repeated measure, the effect of our 10th grade measure is more conservative than it would be if measured alone. Nonetheless, a one standard deviation increase in 10th grade perceived mathematics ability corresponds to a 1.62 times higher risk of staying in the natural sciences as compared to never entering these fields in college. Mathematics ability beliefs in 12th grade are also positively associated with switching into natural science majors, among those not initially intending them.

Additionally, we examined students' choice of postsecondary majors two years after secondary school. We estimated simple models with demographic characteristics and built up to our full model which included ability beliefs. Final models included interaction terms assessing gender differences in how ability beliefs influence major selection. As we added explanatory variables to the model, women's chances of majoring in PEMC—while still considerably smaller than men's—increased. Interestingly, gender strongly influences students' choice of both PEMC and health sciences majors, in opposite directions. Specifically, men have a 3.60 times higher risk of majoring in PEMC sciences as compared to women and a 0.74 times lower risk than women of majoring in health fields. Race/ethnicity also influenced students' choice of scientific majors, in nuanced directions which we are examining further in a forthcoming paper (Nix and Perez-Felkner, 2016). Other than gender and race/ethnicity, advanced science course completion in secondary school was the most significant predictor of majoring in PEMC fields. These reported results are each highly significant (*p* < 0.001). With respect to ability beliefs, all domain-specific beliefs were found significant (*p* < 0.05 or smaller), even verbal (*p* < 0.000). Of these, 12th grade perceived ability under challenge had the strongest predictive influence. We interpret this finding further below.

To add clarity of interpretation, we report these differences using predicted probabilities generated from our estimates, using the margins commands in Stata 14^3^. All other predictors at their means, our models indicate women have a 4.7% chance of declaring PEMC majors as compared to 14.9% of men^4^. All else equal then, being female decreases the students' probability of majoring in PEMC scientific fields by 10.2% points.

How do women and men's chances vary depending on their ability beliefs? Starting with girls: 12th grade girls with the most negative perceptions had a 1.8% chance of choosing PEMC majors; those girls with the most positive perceptions of their mathematics ability under challenge had a 5.6% chance, all else being equal. In other words, girls' likelihood of majoring in PEMC is 3.1 times greater at the highest value of 12th grade mathematics ability under challenge as compared to the lowest value. Turning to boys, those with the most positive perceptions had a 19.1% chance, 2.8 times higher than those with the most negative perceptions (6.7%), all else being equal. While men have the higher raw increase, the rate of increase is a more informative measure. Similar albeit smaller effects are found for 10th grade perceived ability and growth mindset, whereby girls' chances of majoring in PEMC range, respectively, from 1.8 to 5.5 and 1.9 to 4.5% as their beliefs increase, all else being equal.

**Figure 3:**
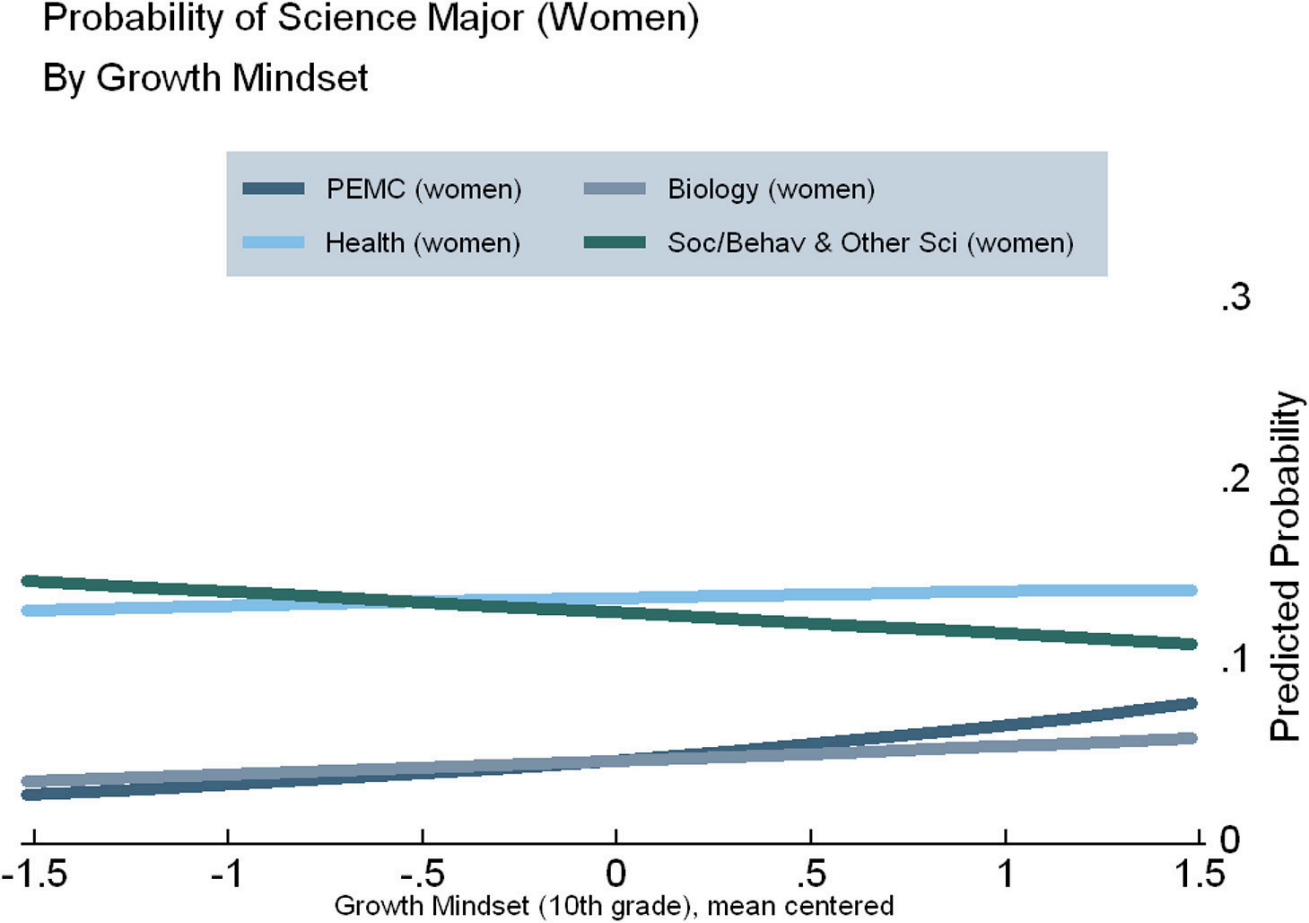
**Predicted probabilities of choosing specific STEM majors, by growth mindset in 10th grade, for girls on the 75th percentile of mathematics ability**.

**Figure 4:**
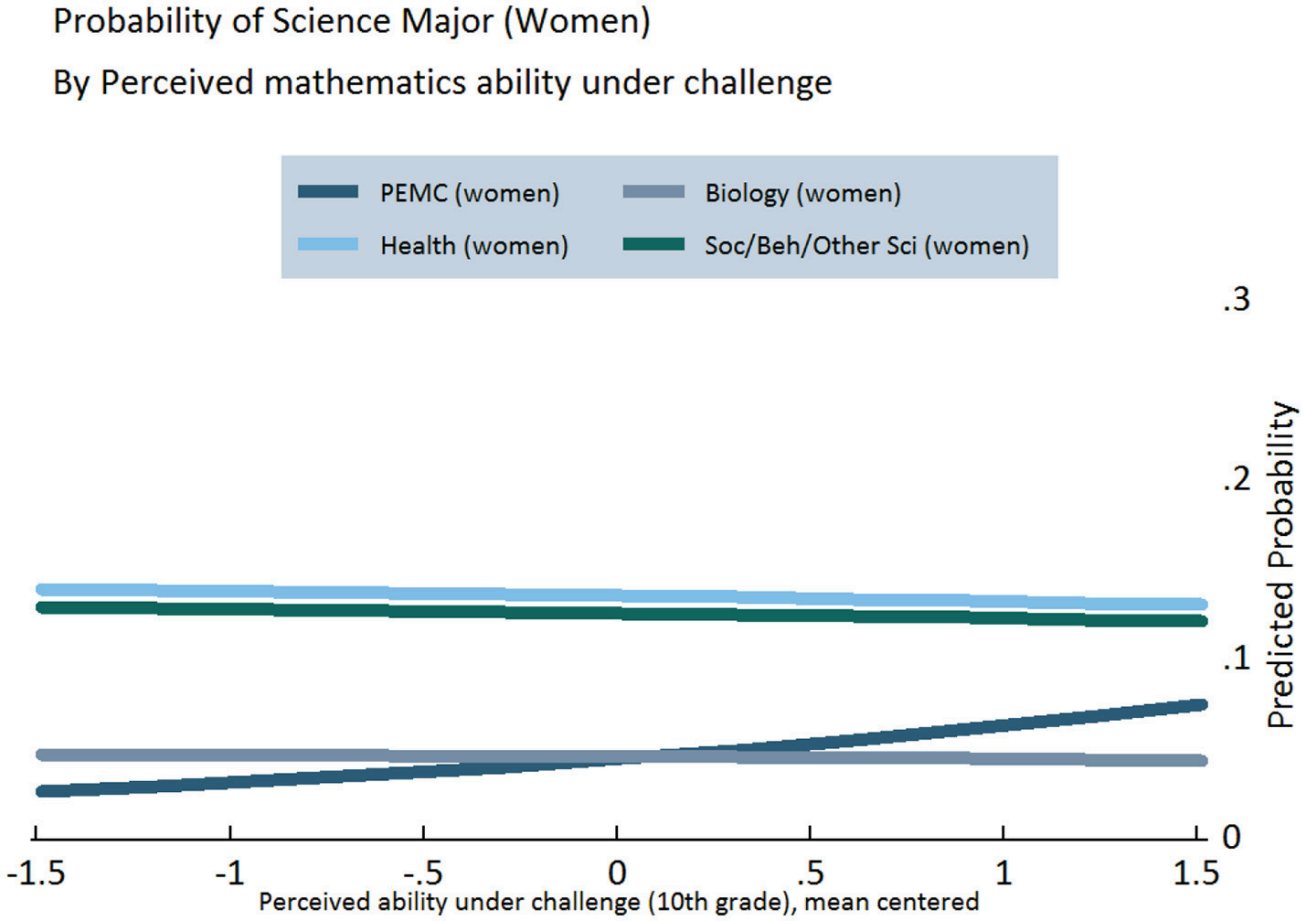
**Predicted probabilities of choosing specific STEM majors, by perceived mathematics ability under challenge in 10th grade, for girls on the 75th percentile of mathematics ability**.

**Figure 5:**
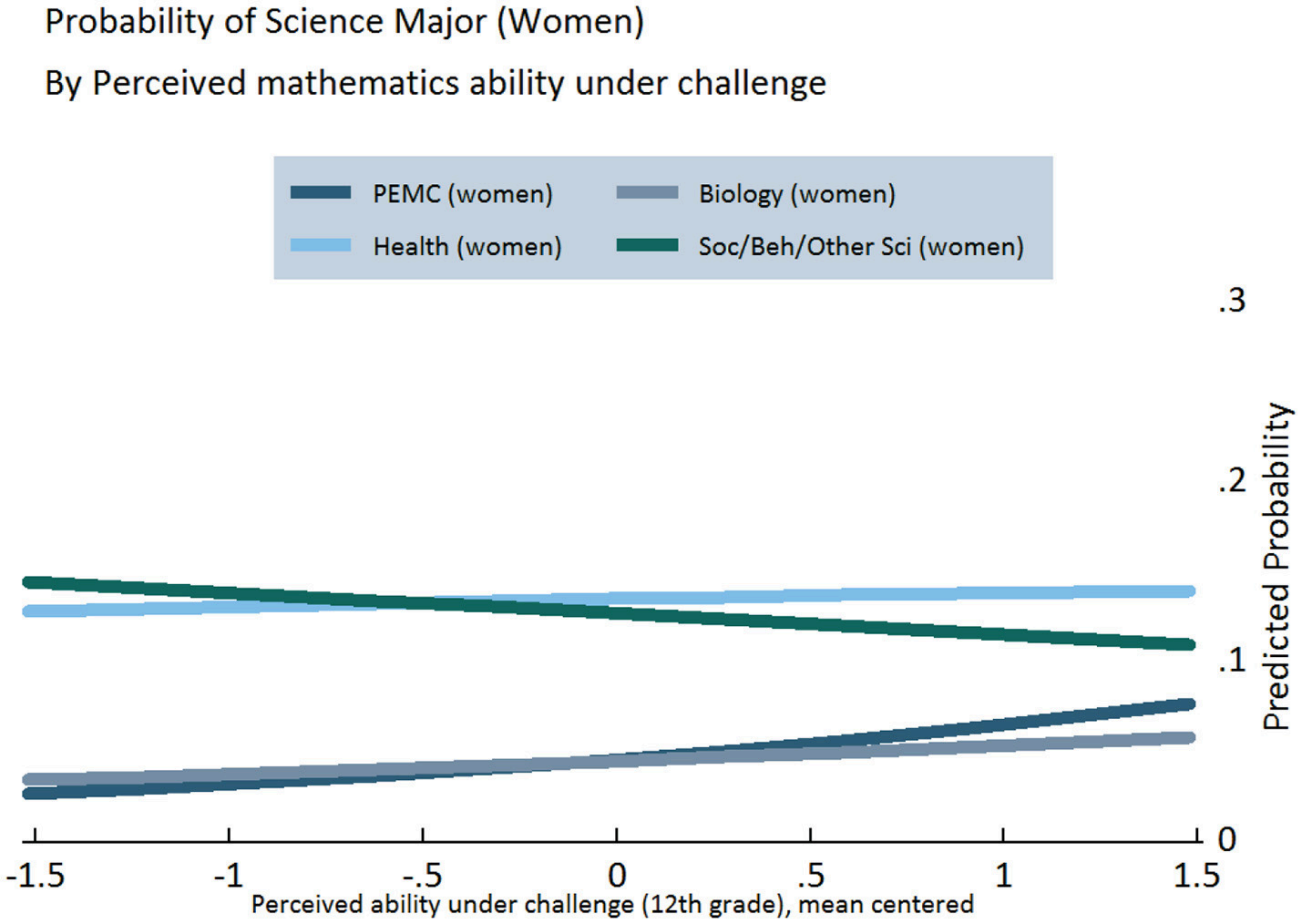
**Predicted probabilities of choosing specific STEM majors, by perceived mathematics ability under challenge in 12th grade, for girls on the 75th percentile of mathematics ability**.

Figures 3–5 display the results of our regression models, showing women's chances of selecting each of our four categories of STEM majors, as predicted by their level of growth mindset (Figure 3) and perceived mathematics ability under challenge in 10th (Figure 4) and 12th grade (Figure 5) (Figures S2, S3). As explained in the Appendix, predicted probabilities are calculated for girls in the 75th percentile of observed mathematics ability in 10th grade, as higher performing students tend to be better positioned to pursue science in postsecondary school^5^. These graphs demonstrate increasing girls' beliefs about their ability with challenging mathematics can raise their probability of majoring in PEMC fields over and above now female-dominated biological science fields, all else being equal. This finding is clearly observed for growth mindset (see Figure 3), positively associated with choosing PEMC majors for girls but negatively associated for boys (see Figure S1 in Supplemental Material). Correspondingly, as 10th grade growth mindset and 12th grade perceived ability under challenge increase, women's probability of majoring in health fields increases over and above that of majoring in social/behavioral and other science fields. Moreover, perceived ability in challenging mathematics is particularly positive and influential for girls, as indicated above and in Figure 4.

Next, we investigated potential gender variation in the relationship between mathematics ability beliefs and scientific major choice. Indeed, we found evidence of one significant interaction, between gender and growth mindset in the choice of health majors. Specifically, believing mathematics ability can be improved through effort (growth mindset) influences men and women differently. While growth mindset does not have a significant effect on women's chances of majoring in health fields, men's chances of majoring in health fields decline by 3.9% points for every one-unit increase in growth mindset^6^. We do not however find evidence indicating boys and girls differ in how any of our mathematics ability belief measures (growth mindset and perceived ability under challenge in 10th and 12th grades) influence choice of PEMC major. Notwithstanding, perceived ability under challenge in mathematics in 12th grade and gender are each significant predictors of majoring in PEMC, even in this otherwise weaker interaction effects model (*p*_male_ < 0.000, *p*_math12_ < 0.009). In other words, perceived ability under challenge in 12th grade clearly and positively affects men and women's chances of entering PEMC fields, as we see also in the results reported above. This influence is in addition to the positive effect of all three ability beliefs as well as high school science course taking, which remains positive and significant.

Figures S1–S3 in the supplementary material represent the predicted probabilities for men, complementing Figures 3–5. The primary differences by gender are as follows. Puzzlingly, Figure S1 indicates that for men at the 75th percentile of observed mathematics ability, those with the lowest growth mindset are the most likely to pursue PEMC fields. By contrast, Figures S2 and S3 show the perceived ability under challenge in mathematics directly increases men's probability of majoring in PEMC fields. Furthermore, these figures represent visually a finding consistent with other contemporary studies (Perez-Felkner et al., 2012; Schneider et al., 2015), that the patterns found in health fields for women correspond to those found in PEMC fields for men. In effect, the gender gap in each—including their close relationship with biology—is mirrored when comparing these figures with their companion figures in the main manuscript.

## Conclusions

Our study explains the relationship between mathematics ability beliefs and girls' and boys' decisions to pursue the most sex-segregated scientific degree fields: physical, engineering, mathematics, and computer sciences (PEMC). Because late secondary school and early college are critical years in U.S. students' decisions about whether to continue studying science (Berryman, 1983; Seymour and Hewitt, 1997; Griffith, 2010), our research uses nationally representative longitudinal data following 10th grade students over a six year period. Indeed, we find mathematics ability beliefs influence students at each stage of our study, from completing more advanced high school science coursework to the undergraduate majors they choose. With respect to students' selection of PEMC majors, our primary outcome of interest, perceived ability under challenge in mathematics is especially influential, for both girls and boys. Our main findings are summarized below.

Mathematics ability beliefs in secondary school vary by gender. We demonstrate a gender difference in mathematics ability perceptions, such that boys hold a growth mindset more often than girls and perceive their mathematics ability to be stronger than do girls, especially in 10th grade. We investigate this relationship further, finding this pattern holds even when controlling for observed mathematics ability and other key predictors. In fact, these gendered patterns hold even for those on the highest ends of the distribution of mathematics ability (boys and girls together), supporting the argument that ability beliefs and their influence cannot be explained by differences in innate talent. We also observe the concerning finding that in this recent cohort of U.S. students attending secondary school in the 2000s, even among the most mathematically talented students, boys remain more confident in their abilities when encountering challenging mathematics. It should then perhaps not be surprising that women remain underrepresented in what are so often colloquially called the “hard” sciences.

Mathematics ability beliefs appear to explain each of our PEMC-related outcomes, at each stage, from completion of advanced science courses in secondary school to earning PEMC undergraduate degrees. Girls' chances of choosing these majors more than tripled as their ability beliefs increased from low to high, even while controlling for key background, secondary school, and postsecondary characteristics. All together, these results suggest enhancing girls' beliefs about their mathematics ability—in particular when encountering challenging math—can have meaningful consequences for their opportunities to pursue fields aligned with their mathematical and scientific talent.

Our findings have practical implications. Because of girls' more negative perceptions of their ability under challenge, difficulty with mathematics may especially steer girls away from scientific majors and careers. However, feeling challenged is a normal and necessary part of learning. Students appear to experience optimal learning when their skill, interest, and challenge are balanced (Schneider et al., 2016). Recent interventions aim to help students' resilience and shift toward more malleable conceptions of intelligence (Good et al., 2003; Bages and Martinot, 2011; Yeager and Dweck, 2012). Moreover, social support—from teachers, mentors, parents, peers—could be a valuable tool to scaffold students through challenges and help girls counter their own and others' lower beliefs about their mathematics ability (Vygotsky, 1980; see Perez-Felkner, 2015).

Moreover, our findings suggest enhancing access to advanced science coursework in secondary and postsecondary school has positive effects on students'—notably girls'—chances of entering PEMC fields in college. While there have been considerable efforts to increase mathematics rigor in U.S. secondary schools, less attention has been paid to science. Some approaches to sustaining girls' interest and engagement through middle and high school include: science camps like SciGirls; recruiting girls to participate in upper level science courses and extracurricular activities; informal science learning experiences; and increasing visibility and access to women scientists both fictional and real. Notably successful efforts at the university level include bias-reducing and social belonging interventions and reducing barriers to enrolling in undergraduate gateway courses to engineering and computing majors (e.g., prior coursework, experience in the field; Corbett and Hill, 2015).

Several reinforcing influences appear to continue signaling to girls that these majors may not be the most appropriate or wise investment of their time. First, there are the persistent stereotypes held by many adults and young people in their life that girls are “just not as good” at math and science, with demonstrated effects among even undergraduate women (Beilock, 2008; Cheryan et al., 2009). Second, these messages, even if well intended, are reinforced by the media and broader society. Perhaps the gap in computing and other scientific fields is attributable to girls' perceived need to be “perfect” rather than brave (Saujani, 2016). Indeed, U.S. boys are more likely to grow up practicing athletic feats and imagining they have superhuman powers while girls often still practice being princesses. While each has their merits, the differentiation is troubling. Socialization research suggests children learn and internalize such cultural messages (Perez-Felkner, 2013), which may influence how girls and boys respond to the inevitable experience of struggling with a difficult mathematics problem or exam—alternately welcoming or avoiding the risk of failure. Third, upper level math and science courses are often optional in school; social network research on course taking patterns indicates girls are particularly likely to follow their same-gender friends into or out of STEM preparatory course work in high school (Riegle-Crumb et al., 2006). Finally, other than medicine, most girls (and boys) know very little about the careers and lives of those working in STEM disciplines. Because of this limited and often times skewed knowledge, there appears to remain a need for initiatives to highlight the potential diversity and joy of these fields, which have not traditionally seemed welcoming to young people from traditionally underrepresented backgrounds, of all genders.

It is difficult to change societal associations between gender and mathematics ability, which boys and girls experience and may internalize early (Eccles, 2005; Jacobs et al., 2005; Gunderson et al., 2012). Nevertheless, these associations have consequences for career aspirations (Correll, 2004) and, as we show here, each step of the secondary and postsecondary pathway to careers in high-need, high-status, and highly sex-segregated physical, engineering, mathematics, and computer sciences (PEMC). Mathematics-intensive science fields are expected to constitute an increasing share of the labor market. While women are the majority of college students, they remain a minority in these majors (DiPrete and Buchmann, 2013). This is a problem. Importantly, beyond the numbers issue, research shows diversity increases the quality of scientific work, helping generate more innovative and influential ideas (Freeman and Huang, 2014). Putting our heads in the sand in response to persistent—and in some cases worsening—gender disparities in science gets us nowhere. Rather, this research implies the need for continued investment in efforts to generate and sustain creative, multi-pronged approaches to help more talented and ambitious girls see themselves as—and become—scientists.

## Author contributions

LP led the writing of this manuscript, a focused review of the paper authored by SN, LP, and KT. LP also designed and conducted the new analyses for this manuscript and revised the manuscript in concordance with reviewer comments. SN engaged in frequent consultation with LP about the analyses, drafted the first round of analyses and Supplementary Material, and contributed to revisions. KT engaged in discussions with the first and second author and contributed considerably to the analytic design and interpretations of the Nix et al. (2015) manuscript we review here. Both SN and KT reviewed and contributed edits to this manuscript.

## Funding

During the writing of this and the prior manuscript, LP and SN were supported by Florida State University's Center for Postsecondary Success, and all three authors have been supported by the same university's Center for Higher Education Research Teaching and Innovation. In addition, LP received funding support as a co-Investigator on National Science Foundation grant #1232139 during the development and completion of the prior manuscript.

### Conflict of interest statement

The authors declare that the research was conducted in the absence of any commercial or financial relationships that could be construed as a potential conflict of interest.

## Footnotes

^1^Correll's (2001) seminal self-assessment study compared students' responses on the following three sets of items from the National Education Longitudinal Study of 1988, alternately about Mathematics or English courses: “Mathematics/English is one of my best subjects,” “I get good marks in Math/English,” and “I have always done well in Math/English.”

^2^As discussed further in the supplementary material, the data do not allow us to separate PEMC and biology fields for the major retention variable. We refer to this combined category as “natural sciences.”

^3^More detailed information on the commands used to generate these probabilities is provided in the supplement.

^4^When we subsequently add our gender^*^ability belief interaction terms, the gender gap in PEMC narrows ever so slightly: women then have a 4.6% chance of majoring in PEMC as compared to 14.7% of men. Both models were found to fit the data effectively (*p* < 0.000), but the simpler model without interactions is stronger [*F*_(120, 80)_ = 9.78 vs. *F*_(145, 55)_ = 8.18]. For this reason, the following results (until line 316) are based on the model without interaction terms.

^5^We considered aligning all of our ability measures to either their sex-specific means or their 75th percentile. To allow the reader to more clearly evaluate the independent effects of each of our mathematics ability beliefs however, all variables are set at their sample means other than gender, observed mathematics ability, and the ability belief under examination (i.e., perceived ability under challenge in mathematics in 10th grade, in 12th grade, and growth mindset).

^6^Recall growth mindset is based on a single item. Therefore, a one-unit increase aligns with students' responses (e.g., from strongly agree to agree).
