# Supplementary material for: Gendered Pathways: How Mathematics Ability Beliefs Shape Secondary and Postsecondary Course and Degree Field Choices
Source: Front Psychol. 2017 Apr 6;8:386. doi: 10.3389/fpsyg.2017.00386 (PMC5382838; doi:10.3389/fpsyg.2017.00386)
Supplement: Supplementary file 1 [file DataSheet1.pdf]

Supplementary Material

Gendered Pathways: How Mathematics Ability Beliefs Shape Secondary and Postsecondary Course and Degree Field Choices

Lara Perez-Felkner[1*], Samantha Nix[1], Kirby Thomas[2]

[1]Center for Postsecondary Success, Department of Educational Leadership and Policy Studies, Florida State University, Tallahassee, FL, USA

[2] Department of Sociology, Florida State University, Tallahassee, FL, USA

*** Correspondence:**
lperezfelkner@fsu.edu

Keywords: STEM, gender, sex segregation, ability beliefs, perceptions, degree fields, mathematics ability, majors

APPENDIX

Data Sample

We used the nationally representative U.S. Education Longitudinal Study of 2002 (ELS), which surveyed 16,200 10th grade students in the 2002 base year of the study (totals rounded for restricted-use data compliance. Our analytic sample was limited to participants who enrolled in postsecondary institutions by 2006, two years after high school. Calibrated bootstrap replicate weights (ELS variables *f2byp1-f2byp200*) and panel survey weighting (ELS variable *f2bywt*) were used to adjust for the stratified sample design and ensure national representativeness. Listwise deletion was used on independent and dependent variables with missing data. With these sample restrictions, the final sample size included 4,450 students. The methodology section of the manuscript (section 3.3) includes additional details.

Variables

A comprehensive list of the variables used in the 2015 article are available in that article's supplementary materials.

The new analyses, Figures 1-5 and S2-S4, discussed in the text, used the following variables.

Dependent Variables: High School Science Pipeline, Major Retention, Declared Major

Science pipeline: We simplified the NCES-generated variable *(f1rscpip)* such that 1 = Chemistry I or Physics I and below; 2 = Chemistry I and Physics I; and 3 = Chemistry II and Physics II.

Major retention: To assess whether students stayed in their intended major two years after high school, we used NCES-generated variables *f2b15* and *f2major2*. *F2b15* reflected students' intended major, through their retrospective response (two years after high school) to the question "When you began your post-secondary education, what field of study did you think you were most likely to pursue?" *F2major2* recorded students' declared majors two years after high school, as designated below. *f2b15* categories and *f2major2* categories differed significantly from one another. In particular, one category for *f2b15* combined natural sciences and mathematics. We therefore collapsed all of the categories for both *f2b15* and *m2major2* into two categories:

- "PEMC + Bio" (F2B15 categories engineering or engineering technology, computer or information sciences, and natural sciences or mathematics; *f2major2* categories biological and biomedical sciences, computer and information sciences or support technology, engineering technologies and technicians, mathematics and statistics, and physical sciences) and
- "All Other Majors."

Major retention was therefore coded as:

- 1 = PEMC and/or Biology major abstainers (All Other Majors → All Other Majors);
- 2 = PEMC and/or Biology stayers (PEMC/Bio → PEMC/Bio);
- 3 = PEMC and/or Biology leavers (PEMC/Bio → All Other Majors); and
- 4 = PEMC and/or Biology newcomers (All Other Majors → PEMC/Bio).

Declared Major: We used ELS variables *f2major2* to indicate major and *f2b22* to indicate whether a major was declared. ELS major variables use the Classification of Instructional Program (CIP) 2-digit coding scheme. Our categories reflect various National Center for Education Statistics reports on what constitutes STEM and non-STEM majors (e.g., Ginder & Mason, 2011).

- 1 = Undeclared/Not in a Degree Program (*f2b22* categories not in a degree program and not yet declared);
- 2 = Non-STEM (*f2major2* categories area, ethnic, cultural, and gender studies; visual and performing arts; business, management, marketing, and related fields; communication, journalism, and communication technology; construction trades; education, English language, literature and letters; family, consumer, and human sciences; foreign languages, literature, and linguistics; legal professions and studies; mechanical and repair technologies and technicians; multi and interdisciplinary studies; parks, recreation, leisure and fitness studies; precision production; personal and culinary services; philosophy, religion and theology; pubic administration and social services; security and protective services; transportation and materials moving; other; and liberal arts, sciences, general studies, and humanities.);
- 3 = PEMC (*f2major2* categories computer/info sciences/support tech, engineering technologies/technicians, mathematics and statistics, and physical sciences);
- 4 = Biological Sciences (*f2major2* category biological and biomedical sciences);
- 5 = Health Sciences (*f2major2* category health professions/clinical sciences);
- 6 = Social/Behavioral and Other Sciences (*f2major2* categories agricultural/natural resources/related, architecture and related services, science technologies/technicians, psychology, social sciences). We combined Social/Behavioral and Other STEM sciences because of small *n*'s in the Other STEM categories and an already complex multinomial logistic model.

Primary Independent Variables: Mathematics Ability Beliefs

With respect to the values of each of the ability beliefs studied, the general, verbal, 10th grade mathematics, and 12th grade mathematics scales were all originally coded such that 1 indicated the least agreement and 4 indicated the most agreement. The growth mindset measure, *bys88a*, had an opposite coding structure. Thus, we reversed the growth mindset coding structure for our analysis, such that each of the variables below ranged from 1 (strongly disagree) to 4 (strongly agree).

Because we were interested in the potential interaction of gender with these ability beliefs in their effect on degree field of study, we created cross-product terms for each of the mathematics ability beliefs that seemed influential in our prior study. Factor analysis was used to create the general, verbal, 10th grade mathematics, and 12th grade mathematics scales. All variables loaded on each factor with eigenvalues over 2.0. Finally, we generated the gender*perceived ability interaction terms.

- **Growth mindset** used ELS variable *bys88a*. This variable measured participants' agreement with the following statement: "Most people can learn to be good at math." The variable was originally coded from 1 to 4 with 1 indicating higher agreement with the statement. We reverse coded the variable to match the coding on the items that made up the perceived ability under challenge measures, which were coded 1 to 4 with 4 indicating higher agreement with the statement. This variable was then standardized to match the perceived ability under challenge measures described below."

The following scales measure **perceived ability under challenge**, in general, verbal, and mathematics domains, and are derived from multiple items, described below. Scales were estimated from the items by domain using factor analysis. All variables loaded on a single factor with a minimum eigenvalue of 1.0. Stata 14's factor analysis function automatically standardizes the values such that the mean = 0 and SD = 1.

- The **general scale** used the following ELS variables: *bys89e*, *bys89j*, *bys89o*, *bys89s*, and *bys89v*. These variables measured participants' agreement with the following statements in the 10th grade:
    - "When I sit myself down to learn something really hard, I can learn it."
    - "When studying, I keep working even if the material is difficult."
    - "When studying, I try to work as hard as possible."
    - "When studying, I try to do my best to acquire the knowledge and skills taught."
    - "When studying, I put forth my best effort."
- The **verbal scale** used the following ELS variables: *bys89c*, *bys89f*, and *bys89m*. These variables measured participants' agreement with the following statements in 10th grade:
    - "I'm certain I can understand the most difficult material presented in English texts."
    - "I'm confident I can understand the most complex material presented by my English teacher."
    - "I'm certain I can master the skills being taught in my English class."
- The **10th grade mathematics scale** used the following ELS variables: *bys89b*, *bys89l*, and *bys89u*. These variables measured participants' agreement with the following statements in 10th grade:
    - "I'm certain I can understand the most difficult material presented in math texts."

- “I’m confident I can understand the most complex material presented by my math teacher.”
    - “I’m certain I can master the skills being taught in my math class.”
- The **12th grade mathematics scale** used the following ELS variables: *f1s18b*, *f1s18c*, and *f1s18e.* These variables measured participants’ agreement with the following statements in 12th grade:
    - “I’m certain I can understand the most difficult material presented in math texts.”
    - “I’m confident I can understand the most complex material presented by my math teacher.”

    “I’m certain I can master the skills being taught in my math class.”

Control Variables

We used the following control variables for the analysis: gender (*f2sex*), race/ethnicity (*byrace_r*), parents’ education (*bypared*), family income (*byincome*), 10th grade standardized mathematics IRT-score (*bytxmirr*), 10th grade standardized reading IRT-score (*bytxrirr*), highest science course taken (*f1rscpip*), 10th grade GPA (*f1ragp10*), high school region (*bycendiv*), high school urbanicity (*byurban*), and college selectivity (*f3tzps1slc*).

Analytic Models

The following models were used to conduct the analyses referenced in the 2015 article, which includes more information on the variables were used in each step of the models. There is only one distinction of note between the 2015 paper and our new analyses: the earlier paper measured 10th grade ability with respect to performance on the most difficult verbal and mathematics ability test sections; the present paper uses broader and commonly used mathematics and reading ability measures. Otherwise, the variables are consistent. Full models are shown below, and correspond respectively to Tables 4-7 from the 2015 article, with the exception of this substitution in the mathematics and reading score variables.[1] The figures generated in this analysis were based on the full model titled “Declared major field (2006), (women= reference).”

$$\begin{aligned} Science\ pipeline\ (2004) &= \beta_0 + \beta_1 female + \beta_2 race/ethnicity + \beta_3 parented + \beta_4 faminc \\ &+ \beta_5 mathscore + \beta_6 readingscore + \beta_7 GPA + \beta_8 HSregion \\ &+ \beta_9 HSurbanicity + \beta_{10} genscale + \beta_{11} verbalscale + \beta_{12} growth \\ &+ \beta_{13} mathscale(10th) \end{aligned}$$

$$\begin{aligned} Retention\ in\ intended\ major\ field\ (2006) &= \beta_0 + \beta_1 female + \beta_2 race/ethnicity + \beta_3 parented + \beta_4 faminc \\ &+ \beta_5 mathscore + \beta_6 readingscore + \beta_7 GPA + \beta_8 Sciencepipeline \\ &+ \beta_9 HSregion + \beta_{10} HSurbanicity + \beta_{11} genscale + \beta_{12} verbalscale \\ &+ \beta_{13} growth + \beta_{14} mathscale(10th) + \beta_{15} mathscale(12th) \\ &+ \beta_{16} collegeselectivity \end{aligned}$$

$$Declared\ major\ field\ (2006), (men = reference)$$

[1] This minor change did not meaningfully effect the magnitude nor significance of study results, when comparing models with prior and current measures.

$$= \beta_0 + \beta_1 female + \beta_2 race/ethnicity + \beta_3 parented + \beta_4 faminc + \beta_5 mathscore + \beta_6 readingscore + \beta_7 GPA + \beta_8 Sciencepipeline + \beta_9 HSregion + \beta_{10} HSurbanicity + \beta_{11} genscale + \beta_{12} verbalscale + \beta_{13} growth + \beta_{14} mathscale(10th) + \beta_{15} mathscale(12th) + \beta_{16} collegeselectivity$$

$$Declared\ major\ field\ (2006),\ (women = reference) = \beta_0 + \beta_1 male + \beta_2 race/ethnicity + \beta_3 parented + \beta_4 faminc + \beta_5 mathscore + \beta_6 readingscore + \beta_7 GPA + \beta_8 Sciencepipeline + \beta_9 HSregion + \beta_{10} HSurbanicity + \beta_{11} genscale + \beta_{12} verbalscale + \beta_{13} growth + \beta_{14} mathscale(10th) + \beta_{15} mathscale(12th) + \beta_{16} collegeselectivity$$

$$Declared\ major\ field\ (2006), with\ interactions = \beta_0 + \beta_1 male + \beta_2 race/ethnicity + \beta_3 parented + \beta_4 faminc + \beta_5 mathscore + \beta_6 readingscore + \beta_7 GPA + \beta_8 Sciencepipeline + \beta_9 HSregion + \beta_{10} HSurbanicity + \beta_{11} genscale + \beta_{12} verbalscale + \beta_{13} growth + \beta_{14} mathscale(10th) + \beta_{15} mathscale(12th) + \beta_{16} male * growth + \beta_{17} male * mathscale(10th) + \beta_{18} male * mathscale(10th) + \beta_{19} collegeselectivity$$

Supplementary Analyses and Figures

To state our findings with clear interpretability, we used Stata 14 commands to generate predicted probabilities for categorical outcomes like major choice. To generate the predicted probabilities explained starting on line 287, about men and women's chances of each major with all other predictors at their mean values, we used the margins command in Stata 14, including its use to derive the average marginal effect increase for men, in interaction with their growth mindset. For the figures that follow and the accompanying discussion (starting around line 293), we used the prtab and prgen from the spost9 legacy suite of user-written commands for use in Stata (Long & Freese, 2014a, 2014b).

Figure S1. Predicted Probabilities of Choosing Specific STEM Majors, by Growth Mindset in 10th Grade, for Boys on the 75th percentile of Mathematics Ability

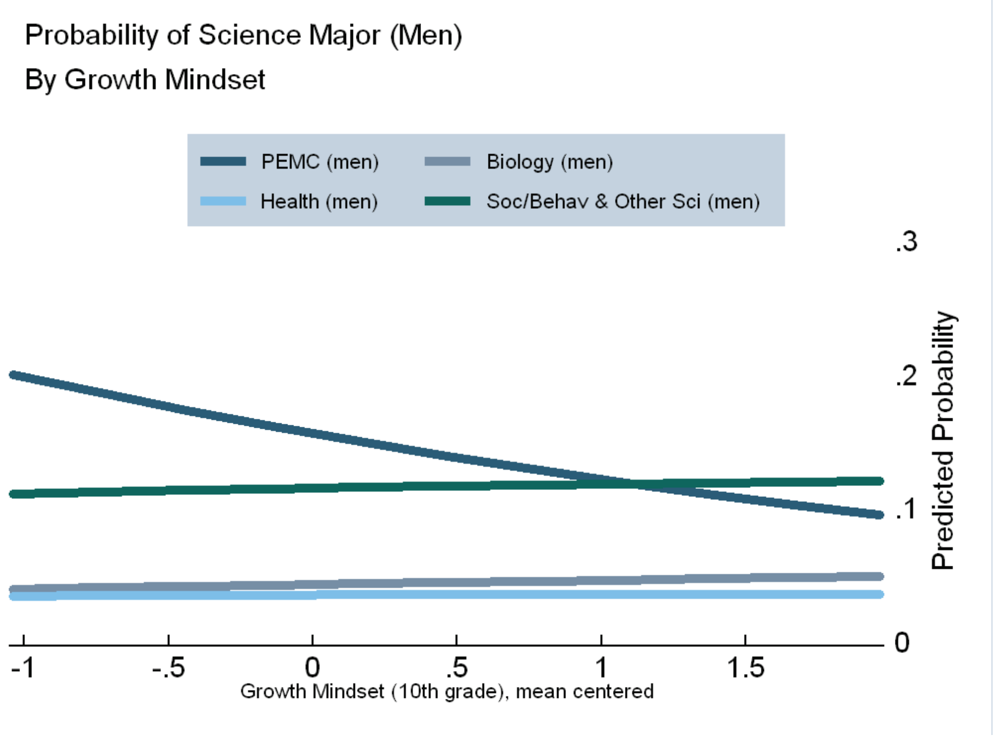

Figure S2. Predicted Probabilities of Choosing Specific STEM Majors, by Perceived Mathematics Ability Under Challenge in 10th Grade, for Boys on the 75th percentile of Mathematics Ability

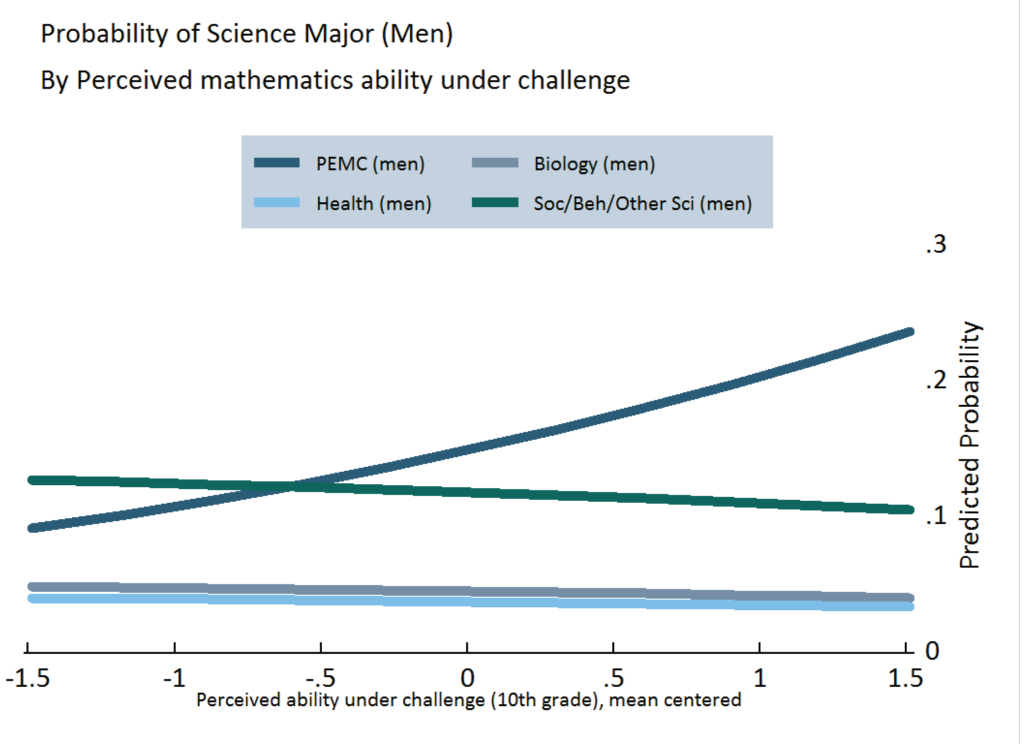

Figure S3. Predicted Probabilities of Choosing Specific STEM Majors, by Perceived Mathematics Ability Under Challenge in 12th Grade, for Boys on the 75th percentile of Mathematics Ability

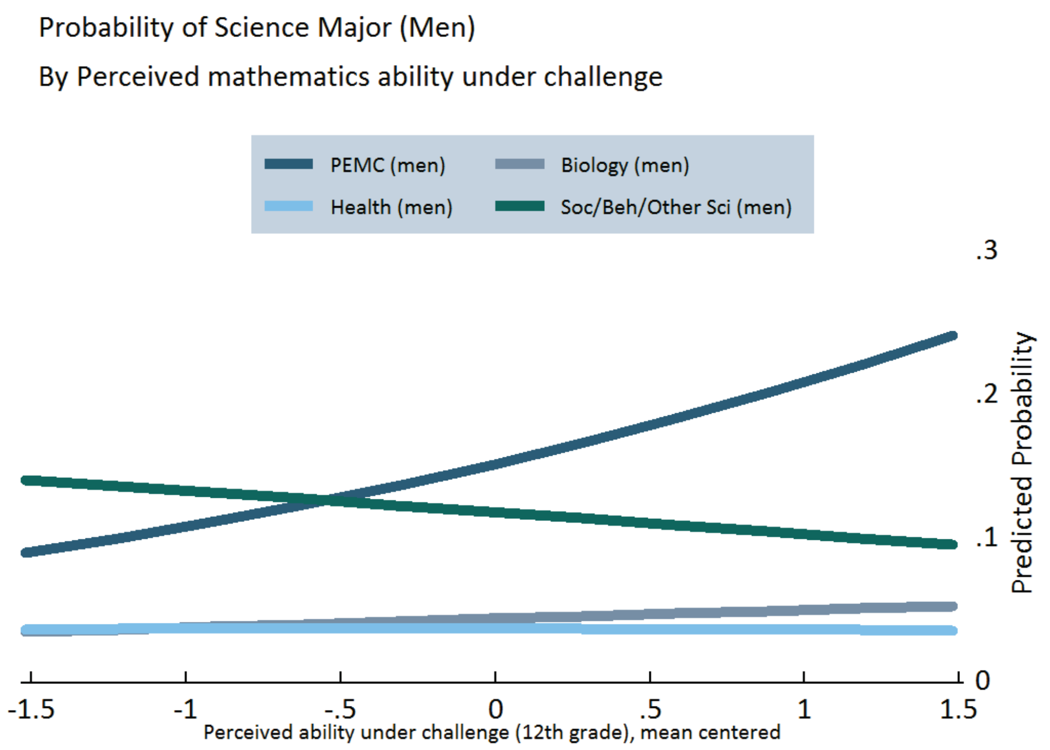

References

Ginder, S. A., & Mason, M. (2011). *Postsecondary Awards in Science, Technology, Engineering, and Mathematics, by State: 2001 and 2009*. Retrieved from Washington, D.C.: http://nces.ed.gov/pubsearch/pubsinfo.asp?pubid=2011226

Long, J. S., & Freese, J. (2014a). *Regression models for categorical dependent variables using Stata* (Third Edition ed.): Stata Press.

Long, J. S., & Freese, J. (2014b). spost9_legacy. Retrieved from www.indiana.edu/~jslsoc/spost.htm
